# A guide to consumer-grade wearables in cardiovascular clinical care and population health for non-experts

**DOI:** 10.1038/s44325-025-00082-6

**Published:** 2025-09-02

**Authors:** Alexandra Jamieson, Timothy J. A. Chico, Siana Jones, Nishi Chaturvedi, Alun D. Hughes, Michele Orini

**Affiliations:** 1 Unit for Lifelong Health and Ageing, UCL, London, UK; 2 Department of Infection, Immunity and Cardiovascular Disease Medical School, University of Sheffield, Sheffield, UK; 3 Department of Biomedical Engineering, King’s College London, London, UK

**Keywords:** Health care, Cardiovascular diseases

## Abstract

Consumer-grade wearables provide an opportunity to understand public health trends, develop risk stratification tools and monitor interventions. This review introduces the most common wearable sensors and describes the health parameters that can be measured using them. We highlight research into the validity and accuracy of these measurements and practical considerations for the use of wearable data. Finally, we provide future perspectives on wearables in cardiovascular clinical practice and population health research.

## Introduction

Use of consumer-grade wearables has grown considerably in recent years^1^. These devices, most commonly in the form of smartwatches, wrist-bands or rings, enable users to access personalised healthcare data and physical activity parameters remotely, continuously and in real-time. In the context of healthcare and population research, wearable technology provides an opportunity to harness data at scale, understand public health trends, develop risk stratification tools and monitor interventions.

Some features of consumer-grade wearables have regulatory board (e.g. United States Food and Drug Administration; US FDA) approval, however, the majority of biometric parameters are derived from sensor signals such as photoplethysmography (PPG) and tri-axial accelerometery and proprietary algorithms which are not available for public scrutiny. Wearable device software and hardware are regularly improved in the form of software updates and new models with an increasing number of features on offer. The proprietary nature and iterative approach in this market makes product comparison and clinical utility difficult to quantify and track in real-time. Furthermore, these devices are engineered and marketed predominantly for individual use and therefore consideration for the practicalities surrounding data acquisition at scale and analysis pipelines is required.

This review aims to provide a non-expert guide to practitioners in clinical care and population health who are considering the use of consumer-grade wearables in cardiovascular healthcare or research settings, with a focus on the following: 1) a general introduction to wearable sensors; 2) the health parameters that can be measured using these sensors; 3) the validity and accuracy of these measurements; 4) practical considerations for the acquisition and use of wearable data at scale and 5) clinical cardiovascular and population health future perspectives.

## What sensors are used in wearables?

**Fig. 1:**
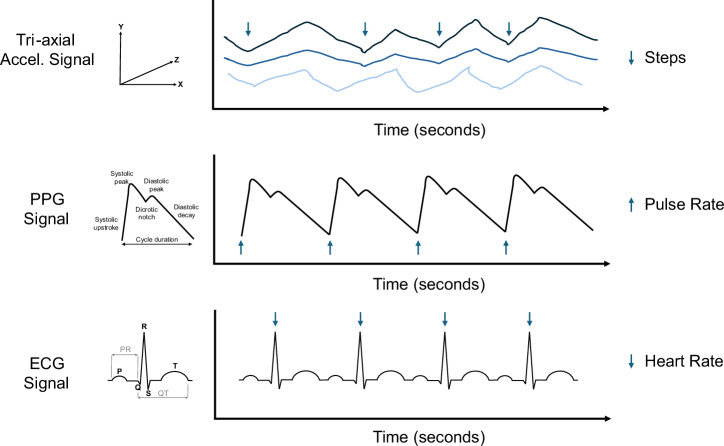
Consumer-grade wearable sensor signals. A schematic representation of three sensor signals commonly used in consumer-grade wearables: tri-axial accelerometery (Accel.), photoplethysmography (PPG) and electrocardiography (ECG).

While a growing number of sensors can potentially be embedded into wearables, most of the physiological data in consumer-grade smartwatches and rings is captured by accelerometery, photoplethysmography (PPG) and electrocardiography (ECG) sensors (Fig. 1).

## Photoplethysmography (PPG)

PPG is a non-invasive optical technique that uses an infrared light source and a detector at the surface of the skin to measure small variations in blood volume. The intensity of the light transmitted through, or reflected from, skin capillaries is proportional to the blood volume and its light absorption^2^. In reflectance PPG, the methodology commonly built into wearables, light is shone from a light emitting diode (LED) on to the skin, and the amount of light reflected back is measured using a photodetector positioned close to the emitting LED^3^. Wearable technology utilises the PPG signal, whose shape is analogous to an arterial pulse wave but its unitless (Fig. 1), to estimate heart rate (HR) and HR variability (HRV)^4^. Other physiological parameters derived from the PPG include respiratory rate^5^, peripheral oxygen saturation^6^ and, more recently, blood pressure (BP)^7,8^.

## Accelerometery

Accelerometers are sensors that detect and measure acceleration forces (the rate of change in velocity). Tri-axial accelerometers can detect changes in movement and orientation in three planes (x; medial-lateral, y; cranial-caudal and Z; anterior-posterior axes) and are widely incorporated into wearables to estimate body position, and several measures of physical activity^9^.

## Electrocardiography (ECG)

The ECG is the gold-standard non-invasive measure of the rhythm of the heart. A standard 12-lead ECG provides a visual representation of the propagation of the electrical impulse through the heart during each cardiac cycle. The variations in the amplitude of the ECG signal over time provide information relating to both HR (e.g., fast or slow) and rhythm (e.g., normal or abnormal). Traditionally, for clinical purposes this method of cardiac monitoring is performed for 10-seconds at rest or can be performed for 24-hours to several days using portable continuous Holter monitors. With advancements in wearable technology, the recording of short ( ~ 30-second) single-lead ECG recordings is now feasible in the most recent devices. These measurements tend to be taken between opposite arms via two electrodes in close proximity to one another, often a positive electrode on the back of a wrist-worn wearable and a negative electrode positioned on the digital screen, to create a bipolar ECG signal similar to lead I of the traditional 12-lead ECG^10,11^.

## Additional Sensors

Wearables are now commonly equipped with many more sensors such as barometers (elevation/altitude), magnetometers (magnetic fields), global positioning systems (GPS; geolocation) and thermometers (temperature), to provide more comprehensive functionality.

## Electrodermal activity (EDA)

Electrodermal activity (EDA) sensors, also known as galvanic skin response sensors use electrodes on the skin to measure subtle variations in electrical conductance. These changes occur as a result of sweat gland activity and are commonly associated with changes in emotional state. In the context of wearables, EDA sensors are often used to infer periods of perceived stress^12,13^.

## Bioelectrical Impedance (BioZ)

Body composition (fat mass, fat free mass, body water content and body fat percentage) can be estimated through the use of bioelectrical impedance analysis (BioZ). BioZ measures the body’s resistance to a low-level electrical current. Traditionally, the integration of this metric with wearable-derived data has been achieved through the use of manufacturer specific compatible bioimpedance scales which can be synchronised to an individual’s wearable health dashboard^14^. More recently, this technology has been integrated into a consumer-grade smartwatch, enabling individuals to estimate body composition by taking a measurement at rest^14^. This is done by placing two fingers from the opposite hand to the watch on designated smartwatch button sensors to administer the microcurrent required for BioZ assessment^15^. Due to its association with tissue hydration status, BioZ has also been used in conjunction with the ECG sensor to predict heart failure decompensation^16,17^ and has been used as part of predictive algorithms in implantable devices^18^.

## Gyroscopes

Gyroscopes are sensors that measure angular velocity (gyration) and can detect very small angular displacements caused by cardiac activity^19^. Gyroscopes are often used in combination with accelerometers in a technique called Gyrocardiography to record cardiac vibration signals in the chest. This technique has been developed to provide insights into the mechanical aspects of the cardiac cycle and detect cardiovascular diseases such as atrial fibrillation (AF) and heart failure using smartphone applications^20^.

## What health parameters can be derived from these sensors and how accurate are they?

Table 1 provides details about commonly used smartwatch, wrist-band and ring (Apple, Fitbit, Garmin, Oura, Polar, Samsung, Withings and Whoop) devices and their biometric features. Table 2 highlights systematic reviews and meta-analyses that have been performed in each of the biometric parameters described in this section along with the wearable manufacturers that were reviewed in each. This table is detailed but not exhaustive.

**Table 1:** Biometric features available in commonly used smartwatch, wrist-band and ring (Apple, Fitbit, Garmin, Oura, Polar, Samsung, Withings and Whoop) wearables

	Apple	Fitbit	Garmin	Oura	Polar	Samsung	Withings	Whoop
**Health metrics**								
Resting HR	Every 5 mins	Every 1 min	Every 15 sec	Every 5 mins	Every 1 sec	Every 5 mins	Every 10 mins	Every 1 sec
HR during activity	Every 1 sec	Every 1 sec	Every 1 sec	Every 1 sec	Every 1 sec	Every 1 sec	Every 1 sec	Every 1 sec
HR sensor	PPG & ECG	PPG	PPG	PPG	PPG	PPG	PPG	PPG
HRV	✓ SDNN	✓ RMSSD	✓ RMSSD SDNN * selected models	✓ RMSSD	✓ RMSSD	✓ SDNN	✓ SDNN * selected models	✓ RMSSD
HRV sensor	PPG & ECG	PPG	PPG	PPG	PPG	PPG	PPG & ECG	PPG
HRV measurements	Sleep & at rest	Sleep	Sleep & Health Snapshot (informs body battery & overnight recovery data)	Sleep	Sleep & training	Sleep & stress tracking	Sleep	Sleep (informs baseline recovery, strain, overall readiness score)
ECG	✓ * Series 4 & later models	✓ * Sense & Charge 5	✓ * Venu2+ onwards, Epix Pro (Gen 2), Fenix 7 pro onwards, quatix 7 Pro, tactix 7, D2 Mach 1 Pro, Enduro 3	×	✓ * Grit X2 Pro, Vantage V3	✓ * Galaxy Watch 3, Galaxy Watch Active 2 & later models	✓ * ScanWatch & Move ECG	×
ECG recording duration	30 s on demand	30 s on demand	30 s on demand	n/a	30 s on demand	30 s on demand	30 s on demand	n/a
ECG reporting	Sinus rhythm Atrial fibrillation Low or high HR ( < 50 or >120 bpm) Inconclusive Poor recording	Sinus rhythm Atrial Fibrillation	Sinus rhythm Atrial Fibrillation	n/a	Average HR HRV Beat to beat interval Pulse arrival time Orthostatic test	Sinus rhythm Atrial Fibrillation	Sinus rhythm Possible Atrial Fibrillation	n/a
FDA clearance for AF detection	✓ ECG & PPG	✓ ECG & PPG	×	×	×	✓ ECG	✓ ECG	×
SpO_2_	✓ Blood Oxygen * Series 6 & later models	✓ SpO_2_ Sensor / Estimated Oxygen Variation * Charge 4 & later models, Sense & Versa series	✓ Pulse Ox (On demand or continuously)	✓ Blood oxygen sensing * Gen 3	✓ SpO2 * Vantage	✓ Blood oxygen * Galaxy Watch 3, Galaxy Watch Active 2 & later models	✓ Oxygen saturation On demand and sleep tracking	✓ Blood oxygen saturation * 4.0
Respiratory rate	✓ Respiratory rate * Series 4 & later models	✓ Breathing rate * Charge 4 & later models, Sense & Versa series	✓ Respiration rate * Venu, Fenix, Forerunner series	✓ Respiratory rate * Gen 2 & onwards	✓ Respiration rate * Vantage	✓ Respiratory rate * Galaxy Watch 3 & onwards	✓ Breathing disturbances	✓ Respiratory rate * 3.0 onwards
Respiratory rate measurements	Sleep	Sleep, rest & activity	Sleep, rest & activity	Readiness & sleep scores	Nightly recharge Serene guided breathing exercise	Sleep analysis	Irregular breathing patterns during sleep	Continuously
Temperature	✓ Wrist temperature * Series 8 & later models	✓ Skin temperature variation * Sense, Versa 3 & Charge 5	✓ Skin temperature * Venu3 onwards, Epix Pro (Gen 2), Fenix 7 pro onwards, quatix 7 Pro, tactix 7, D2 Mach 1 Pro, Enduro 3	✓ Body temperature * Gen 3	✓ Nightly skin temperature	✓ Skin temperature * Galaxy Watch 5	✓ Body temperature * Scanwatch 2	✓ Skin temperature * 3.0 onwards
Stress / readiness score	×	✓ Stress management score / daily readiness score	✓ Stress tracking / body battery	✓ Readiness score / daytime stress / resilience	✓ Nightly recharge	✓ Stress monitoring	✓ Stress level	✓ Stress score / day strain / recovery
Stress/ readiness score based on	n/a	EDA, HRV & sleep	HRV, sleep & activity	HRV, sleep, recovery metrics	HRV & sleep	HRV & other biometrics	HRV	HRV & HR data
Blood pressure	× Compatible with external BP cuffs	×	×	×	×	✓ * requires periodic calibration with a traditional BP cuff * region dependent * Galaxy Watch 3, Galaxy Watch Active 2 & later models	× Compatible with external BP cuffs (Withings BPM Connect)	×
Body composition	×	× * From compatible smart scales (Fitbit Aria)	× * From compatible smart scales (Garmin Index Scale)	×	×	✓ Body fat %, skeletal muscle, basal metabolic rate & body water. * Galaxy Watch 4 & onwards	× * From compatible smart scales (Withings Body+ and Body Cardio)	×
Energy expenditure	✓ Active & total calories	✓ Active & resting calories	✓ Total calories	✓ Calories burned	✓ Total calories burned & calorie tracking for workouts	✓ Calories burned during activities & during the day	✓ Calories burned	✓ Calories burned
Women’s health	✓ Digital diary with predictions	✓ Digital diary with predictions	✓ Digital diary with predictions	✓ Digital diary with predictions	×	✓ Digital diary with predictions	✓ Digital diary	✓ Digital diary
**Activity metrics**								
Step count	✓	✓	✓	✓	✓	✓	✓	✓
Distance	✓ via step count & GPS	✓ via step count & GPS	✓ via step count & GPS	✓ via step count	✓ via step count & GPS	✓ via step count & GPS	✓ via step count & GPS	✓ via activity data
Built in GPS	✓ most models	✓ most models	✓ most models	× location access can be granted via mobile phone app	✓ most models	✓ most models	✓ most models	× location access can be granted via mobile phone app
Floors climbed	✓ 3 m (10 feet)	✓ 3 m (10 feet)	✓ 3 m (10 feet)	×	✓ * Vantage & Grit series	✓ 3 m (10 feet)	×	×
Auto. activity recognition	✓	✓ SmartTrack	✓ Auto Activity Start	×	✓ Automatic Training Detection	✓ Auto Detect Activity	✓ Automatic Workout Detection	✓
CRF	✓ VO_2_max (Cardio Fitness Level)	✓ Cardio Fitness Score	✓ VO_2_max	✓ Cardio Capacity	✓ Fitness test for VO_2_max	✓ VO_2_max	✓ Cardio Fitness	✓ Strain Score
CRF estimate based on	HR & activity data from outdoor walking or running	HR & activity data	HR & activity data from outdoor activities (walking, running, cycling)	Anthropometric data & walking test	HR during specific activities	During specific workouts	HR & activity data	Workout intensity (indirect cardiovascular fitness)
CRF units	ml/kg/min	ml/kg/min	ml/kg/min	ml/kg/min	ml/kg/min	ml/kg/min	ml/kg/min	Score
**Sleep**								
Time spent asleep	✓	✓	✓	✓	✓	✓	✓	✓
Sleep stages	✓ REM, Core & Deep	✓ Light, deep & REM	✓ Light, deep & REM	✓ Light, deep & REM	✓ Light, deep & REM	✓ Light, deep & REM	✓ Light, deep & REM	✓ Light, deep & REM
**Technical specifications**								
Bluetooth connectivity	✓	✓	✓	✓	✓	✓	✓	✓
iOS requirements	iPhone 6 s or later with iOS 14 or later	iOS 14 or later	iOS 12 or later	iOS 12 or later	iOS 12 or later	May not support all features with iOS	iOS 12 or later	iOS 12 or later
Android requirements	Not compatible	Android 8.0 or later	Android 6.0 or later	Android 8.0 or later	Android 6.0 or later	Android 6.0 or later	Android 6.0 or later	Android 6.0 or later
Battery life	~3 days	~5 days	~5 days	4–7 days	5–14 days	1–2 days	30 days	4–5 days

*HR* heart rate, *PPG* photoplethysmography, *ECG* electrocardiography, *RMSSD* root mean square of successive differences, *SDNN* standard deviation of the normal sinus beat intervals, *HRV* heart rate variability, *FDA* Food & Drug administration, *AF* atrial fibrillation, *EDA* electrodermal activity, *GPS* geolocation positioning system, *CRF* cardiorespiratory fitness, *REM* rapid eye movement.

**Table 2:** Highlighted publications organised by wearable biometric parameter

	First Author [Reference]	Year	Title	Wearable Manufacturer
**Health metrics**				
**HR**				
Systematic review/ meta-analysis	Chan^135^	2022	Novel wearable and contactless heart rate, respiratory rate, and oxygen saturation monitoring devices: a systematic review and meta-analysis.	Apple Cardiacsense Everion Fitbit Samsung Wavelet
Systematic review/ meta-analysis	Chevance^136^	2022	Accuracy and precision of energy expenditure, heart rate, and steps measured by combined-sensing fitbits against reference measures: systematic review and meta-analysis.	Fitbit
Systematic review/ meta-analysis	Fuller^33^	2020	Reliability and validity of commercially available wearable devices for measuring steps, energy expenditure, and heart rate: systematic review.	Apple Fitbit Garmin Mio Polar Samsung Withings Xiaomi
Systematic review/ meta-analysis	Germini^137^	2022	Accuracy and acceptability of wrist-wearable activity-tracking devices: systematic review of the literature.	Apple Basis Fitbit Garmin Polar Huawei Jawbone Withings Xiaomi
Systematic review/ meta-analysis	Irwin^138^	2022	Systematic review of Fitbit Charge 2 validation studies for exercise tracking.	Apple Empatica Fitbit Honor Huawei Polar Samsung Wavelet Health Xiaomi
Systematic review/ meta-analysis	Koerber^139^	2022	Accuracy of heart rate measurement with wrist-worn wearable devices in various skin tones: a systematic review.	Apple Fitbit Garmin Mio Alpha
Systematic review/ meta-analysis	Zhang^24^	2020	Validity of wrist-worn photoplethysmography devices to measure heart rate: a systematic review and meta-analysis.	Apple Basis Peak Empatica Fitbit Garmin Microsoft Mio Omron Philips Polar PulseOn Samsung Tempo TomTom Wavelet
**HRV**				
Systematic review/ meta-analysis	Board^140^	2016	Validity of telemetric-derived measures of heart rate variability: a systematic review.	Polar Suunto
Systematic review/ meta-analysis	Dobbs^141^	2019	The Accuracy of Acquiring Heart Rate Variability from Portable Devices: A Systematic Review and Meta-Analysis.	Polar Suunto
Systematic review/ meta-analysis	Georgiou^47^	2018	Can Wearable Devices Accurately Measure Heart Rate Variability? A Systematic Review.	4IIII 60beat BlueLeza Cardiosport Carre Technologies Cositea Empatica Garmin Mad Apparel Medronic Mio Polar Qardio Sony Sunnto Wahoo Fitness Whoop
**ECG/Arrhythmia detection**				
Systematic review/ meta-analysis	Belani^142^	2021	Accuracy of detecting atrial fibrillation: a systematic review and meta-analysis of wrist-worn wearable technology.	Apple Kardiaband Samsung
Systematic review/ meta-analysis	Giebal^143^	2019	Accuracy of mHealth devices for atrial fibrillation screening: systematic review.	Apple Fitbit Polar
Systematic review/ meta-analysis	Hermans^144^	2022	Mobile health solutions for atrial fibrillation detection and management: a systematic review.	Apple Empatica Fitbit Honor Huawei Polar Samsung Wavelet Health Xiaomi
Systematic review/ meta-analysis	Koerber^139^	2022	Accuracy of heart rate measurement with wrist-worn wearable devices in various skin tones: a systematic review.	Apple Fitbit Garmin Mio Alpha
Systematic review/ meta-analysis	Lopez^145^	2021	Mobile health applications for the detection of atrial fibrillation: a systematic review.	Empatica Honor Huawei Wavelet Health
Systematic review/ meta-analysis	Nazarian^146^	2021	Diagnostic accuracy of smartwatches for the detection of cardiac arrhythmia: systematic review and meta-analysis.	Apple Empatica Huawei Huami Samsung Wavelet wristband
**SpO**_**2**_				
Systematic review/ meta-analysis	Windisch^64^	2023	Accuracy of the Apple Watch Oxygen Saturation Measurement in Adults: A Systematic Review.	Apple
**Respiratory Rate**				
Systematic review/ meta-analysis	Chan^135^	2022	Novel wearable and contactless heart rate, respiratory rate, and oxygen saturation monitoring devices: a systematic review and meta-analysis.	Apple Cardiacsense Everion Fitbit Samsung Wavelet
**Stress**				
Systematic review/ meta-analysis	Hickey^116^	2021	Smart Devices and Wearable Technologies to Detect and Monitor Mental Health Conditions and Stress: A Systematic Review.	Apple Bodymonitor Empatica Polar Samsung
**Blood or pulse pressure**				
Systematic review/ meta-analysis	Islam^98^	2022	Wearable cuffless blood pressure monitoring devices: a systematic review and meta-analysis.	B-pro Checkme Freescan SeismoWatch T2-Mart
Perspective	Schutte^95^	2024	Wearable cuffless blood pressure tracking: when will they be good enough?	
**Energy expenditure**				
Systematic review/ meta-analysis	Chevance^136^	2022	Accuracy and precision of energy expenditure, heart rate, and steps measured by combined-sensing fitbits against reference measures: systematic review and meta-analysis.	Fitbit
Systematic review/ meta-analysis	Evenson^147^	2015	Systematic review of the validity and reliability of consumer-wearable activity trackers.	Fitbit Jawbone
Systematic review/ meta-analysis	Feehan^148^	2018	Accuracy of fitbit devices: systematic review and narrative syntheses of quantitative data.	Fitbit
Systematic review/ meta-analysis	Fuller^33^	2020	Reliability and validity of commercially available wearable devices for measuring steps, energy expenditure, and heart rate: systematic review.	Apple Fitbit Garmin Mio Polar Samsung Withings Xiaomi
Systematic review/ meta-analysis	Germini^137^	2022	Accuracy and acceptability of wrist-wearable activity-tracking devices: systematic review of the literature.	Apple Basis Fitbit Garmin Polar Huawei Jawbone Withings Xiaomi
Systematic review/ meta-analysis	Henriksen^149^	2020	Measuring physical activity using triaxial wrist worn polar activity trackers: a systematic review.	Polar
Systematic review/ meta-analysis	Leung^150^	2022	A meta-analysis of Fitbit devices: same company, different models, different validity evidence.	Fitbit
Systematic review/ meta-analysis	O’Driscoll^151^	2020	How well do activity monitors estimate energy expenditure? A systematic review and meta-analysis of the validity of current technologies.	Apple Basis Beurer Epson ePulse Fitbit Garmin Jawbone LifeCheck Microsoft Mio Misft Nike Polar Samsung SenseWear TomTom Vivago Withings
**Activity metrics**				
**Step count & distance covered**				
Systematic review/ meta-analysis	Chevance^136^	2022	Accuracy and precision of energy expenditure, heart rate, and steps measured by combined-sensing fitbits against reference measures: systematic review and meta-analysis.	Fitbit
Systematic review/ meta-analysis	Evenson^147^	2015	Systematic review of the validity and reliability of consumer-wearable activity trackers.	Fitbit Jawbone
Systematic review/ meta-analysis	Feehan^148^	2018	Accuracy of fitbit devices: systematic review and narrative syntheses of quantitative data.	Fitbit
Systematic review/ meta-analysis	Fuller^33^	2020	Reliability and validity of commercially available wearable devices for measuring steps, energy expenditure, and heart rate: systematic review.	Apple Fitbit Garmin Mio Polar Samsung Withings Xiaomi
Systematic review/ meta-analysis	Germini^137^	2022	Accuracy and acceptability of wrist-wearable activity-tracking devices: systematic review of the literature.	Apple Basis Fitbit Garmin Polar Huawei Jawbone Withings Xiaomi
Systematic review/ meta-analysis	Henriksen^149^	2020	Measuring physical activity using triaxial wrist worn polar activity trackers: a systematic review.	Polar
Systematic review/ meta-analysis	Irwin^138^	2022	Systematic review of Fitbit Charge 2 validation studies for exercise tracking.	Apple Empatica Fitbit Honor Huawei Polar Samsung Wavelet Health Xiaomi
Systematic review/ meta-analysis	Kenyon^152^	2013	Validity of pedometers in people with physical disabilities: a systematic review.	Yamax Dig-Walker SW
**Physical activity (PA)**				
Systematic review/ meta-analysis	Chan^135^	2022	Reporting adherence, validity and physical activity measures of wearable activity trackers in medical research: A systematic review.	ActiGraph Fitbit Axivity
Systematic review/ meta-analysis	Feehan^148^	2018	Accuracy of fitbit devices: systematic review and narrative syntheses of quantitative data.	Fitbit
Systematic review/ meta-analysis	Germini^137^	2022	Accuracy and acceptability of wrist-wearable activity-tracking devices: systematic review of the literature.	Apple Basis Fitbit Garmin Polar Huawei Jawbone Withings Xiaomi
Systematic review/ meta-analysis	Henriksen^149^	2020	Measuring physical activity using triaxial wrist worn polar activity trackers: a systematic review.	Polar
**Cardiorespiratory fitness**				
Systematic review/ meta-analysis	Molina-Garcia^86^	2022	Validity of Estimating the Maximal Oxygen Consumption by Consumer Wearables: A Systematic Review with Meta-analysis and Expert Statement of the INTERLIVE Network.	Garmin Fitbit Polar
**Sleep**				
Scoping review	Birrer^153^	2024	Evaluating reliability in wearable devices for sleep staging.	Actical ActiGraph Actiwatch Apple AW-64 Basis Empatica Fitbit Garmin GeneActiv GTX3+ Jawbone Motionlogger MyCadian Oura Polar Whoop Zulu
Systematic review/ meta-analysis	Evenson^147^	2015	Systematic review of the validity and reliability of consumer-wearable activity trackers.	Fitbit Jawbone
Systematic review/ meta-analysis	Feehan^148^	2018	Accuracy of fitbit devices: systematic review and narrative syntheses of quantitative data.	Fitbit
Systematic review/ meta-analysis	Haghayegh^111^	2019	Accuracy of Wristband Fitbit Models in Assessing Sleep: Systematic Review and Meta-Analysis.	Fitbit
Systematic review/ meta-analysis	Imtiaz^154^	2021	A Systematic Review of Sensing Technologies for Wearable Sleep Staging.	Apple Basis Fitbit Microsoft Oura Samsung Whoop Zulu
Review	Rentz^155^	2021	Deconstructing Commercial Wearable Technology: Contributions toward Accurate and Free-Living Monitoring of Sleep.	
Systematic review/ meta-analysis	Schyvens^108^	2024	Accuracy of Fitbit Charge 4, Garmin Vivosmart 4, and WHOOP Versus Polysomnography: Systematic Review.	Fitbit Garmin Whoop
Systematic review/ meta-analysis	Scott^156^	2020	A systematic review of the accuracy of sleep wearable devices for estimating sleep onset.	Actiwatch Fitbit GT3X+ Jawbone Sleepwatch Somno Withings
**Women’s Health**				
Systematic review/ meta-analysis	Lyzwinski^120^	2024	Innovative approaches to menstruation and fertility tracking using wearable reproductive health technology: systematic review.	Ava Oura

A living umbrella review of systematic reviews evaluating the accuracy of consumer-grade technologies in health measurement can be found elsewhere^21^.

## Resting heart rate (HR) and HR tracking

HR is modulated by the autonomic nervous system (ANS) and can be modified by several physiological and environmental factors. HR changes in response to many physiological and medical stimuli, such as exercise, anxiety, pregnancy, physical fitness, and cardiovascular and non-cardiovascular diseases. A chronically elevated resting HR is a strong independent risk factor for all-cause mortality and for adverse outcomes in individuals with cardiovascular disease^22,23^.

The accuracy of PPG estimation of HR has been widely documented with validation performed against reference ECG measurements. At rest, wearables are widely considered to measure HR accurately, with mean absolute errors (AE) in the region of 2 beats per minute (bpm), mean absolute percentage errors (MAPE) reported as less than 10% and correlations between the devices and reference methods consistently reported as moderate to excellent^24–32^.

A systematic review of the reliability and validity of commercially available wearables (Fitbit, Apple Watch, Samsung and Garmin) was performed for the measurement of HR (Table 2)^33^. A total of 29 studies examined wearable device HR measurements compared with reference measures including ECG, Polar chest straps and pulse oximetry. Of the 177 comparisons, 100 (56.5%) were within ±3% measurement error, 44 (24.9%) were below -3% measurement error and 33 (18.6%) were above 3% measurement error, with a slight tendency to underestimate HR^33^.

However, the accuracy of HR measurement in wearables is known to decline during physical activity. In addition to activity intensity, the activity type and specifically arm movement during the activity have been shown to influence the accuracy of HR measurements^34–36^. We recently observed excellent accuracy in measuring HR at rest, and during recovery (MAPE ≤3%), in both Garmin and Fitbit devices, but accuracy worsened during peak exercise^37^. MAPE was similar to rest and recovery during peak exercise, however, the limits of agreement widened due to an increase in the number of outliers ( ~ 7% for Garmin and ~ 12% for Fitbit)^37^. Contact pressure and sweat have also been shown to impact accuracy^38^.

## Heart rate variability (HRV) and pulse rate variability (PRV)

HRV is a measure of the variation in the time interval between each successive heartbeat, specifically the variation in the duration of consecutive R-R intervals on an ECG. HRV is considered a non-invasive ANS marker representing the balance between the sympathetic and parasympathetic branches^39^. Low HRV is associated with cardiovascular disease, diabetes mellitus, hypertension, arrhythmia and all-cause mortality^40^ and conversely, optimal HRV is associated with health and resilience^41^. There are several HRV parameters that can be measured from an ECG which are grouped primarily into time domain and frequency domain markers^42^, but more complex markers have been proposed^43^. Time-domain measurements quantify HRV over a period of time (e.g., 2 min to 24 h) and include the root mean square of successive differences (RMSSD) and the standard deviation of normal-to-normal intervals (SDNN). In contrast, frequency domain metrics measure the signal in various bands of frequency and include high frequency power and low frequency power.

In the context of wearables, ‘HRV’ measurements may be obtained using an ECG sensor, PPG sensor or both. When measured using PPG, the most appropriate metric to use would be pulse rate variability^44,45^, which is derived from consecutive pulses recorded in the wrist or finger and not from consecutive R-R intervals on an ECG. Despite presenting some differences related to the pulse arrival time, i.e., the interval between the R-wave in the ECG and the onset of the PPG pulse, HRV from ECG and PPG have been shown to be similar, even in dynamic conditions^45^. Smartwatches measure the ECG for a short period of time, typically 30 s, from which only measures of ultra-short HRV can be derived^22,46^.

Pulse rate variability has been shown to correlate with HRV during rest and during autonomic challenges^45^. According to a recent review, mainly focusing on measurements derived at rest, the correlation between ECG and wearable derived HRV ranged from very good to excellent at rest and declined progressively as exercise intensity increased (Table 2)^47^.

In a recent validation study of PPG derived HRV (RMSSD and SDNN) using Garmin’s health snapshot, we observed a strong correlation (between 0.82 and 0.89) between Garmin and reference ECG HRV^32^.

## Arrhythmia and AF detection

An arrhythmia refers to an abnormality of the heart’s rhythm in which the heart may beat too slowly, too quickly or irregularly. AF is the most common serious arrhythmia, and refers to an irregular heart rhythm in which uncoordinated electrical activation in the top chambers of the heart (the atria) can impair cardiac efficiency. Although many people with AF are not aware of it, AF can also cause disabling symptoms of an awareness of an unusual heartbeat, breathlessness, dizziness and fatigue. AF affects approximately 59 million individuals worldwide and is associated with an increased risk of blood clots and stroke^48^. Early detection of AF allows for the prompt implementation of patient management and treatment such as medication, or interventions (such as ablation or cardioversion) as well as risk reduction for the development of AF complications such as stroke and heart failure. Wearables can detect AF through both ECG and PPG sensors^49–51^. Currently, other arrhythmias (e.g., atrial or ventricular tachycardia) or premature contractions (called ectopic beats) are not usually detected by consumer-grade smartwatches or rings, despite this may potentially contribute to improving risk assessment^52^.

Wrist-worn devices have been shown to have excellent diagnostic accuracy in AF diagnosis based on a systematic review and meta-analysis of 28 studies (*n* = 13,463, area under the ROC curve of 0.97 (95% CI: 0.94,0.99); Table 2)^53^.

The BASEL wearable study reported that the sensitivity and specificity for AF detection were 85% and 75% for the Apple Watch 6, 85% and 75% for the Samsung Galaxy Watch 3, 58% and 75% for the Withings Scanwatch and 66% and 79% for the Fitbit Sense, respectively^54^. The author’s reported that in a clinical setting, manual review of tracings is required in about one-fourth of cases.

Wearable models from Apple, Fitbit, Samsung and Withings have been cleared by the US FDA for pre-diagnostic AF detection that are not intended for clinical decision-making.

## Cardiac intervals

Cardiac intervals such as the QT interval, which measures the duration of ventricular repolarisation, or the PR interval, which measures the duration of atrial activation, carry important diagnostic and prognostic value (Fig. 1). Recent studies have shown that cardiac intervals derived from smartwatch ECGs show moderate to strong correlation with standard medical-grade ECGs^55–57^, however, these are not currently measured by consumer-grade wearables.

It was noted in a 2023 review that only two commercially available devices (Apple Watch and Withings ScanWatch) had been adequately compared to 12-lead ECG measurements with respect to QTc measurements^56^. In 177 patients (56%), the Withings ScanWatch automated algorithm was able to automatically measure QTc with a mean difference of 6.6 ms [Limits of Agreement; LoA: −59, 72 ms] compared to manual measurements. The authors concluded that the Withings ScanWatch tends to underestimate the QTc interval in line with others^56,58^.

In another study, adequate QT measurements were observed in 85% of patients when the smartwatch was worn in the standard wrist position^55^.

## Respiratory rate

Respiratory rate refers to the number of breaths taken per minute, and like HR is affected by a wide range of physiological and medical conditions^59,60^. Respiratory rate can be estimated by wearables through the analysis of subtle changes in the ECG or PPG signal that occur due to respiratory modulation; including baseline wander of the signal, changes in the amplitude of the signal and the frequency of the signal^5,61^.

The accuracy of the respiratory rate estimation during sleep using the Samsung Galaxy Watch compared to polysomnography has been investigated in 195 individuals with varying degrees of obstructive sleep apnoea (OSA)^62^. OSA is a sleep disorder characterised by periods of partial or complete closure of the airway resulting in reduced and irregular respiratory rate during sleep. The root mean squared error (RMSE) of the average overnight and continuous respiratory rate measurements were 1.13 bpm and 1.62 bpm, respectively, showing a small bias of 0.39 bpm and 0.37 bpm, respectively^62^. In participants with normal-to-moderate OSA, average overnight and continuous respiratory rate measurements were at least 90% accurate^62^. For patients with severe OSA, accuracy decreased to 79.5% and 75.8%, respectively^62^. Recently, Samsung and Apple watches offer the possibility to detect increased risk for OSA, however little validation data is currently available.

## Pulse oximetry (SpO_2_)

Oxygen saturation is a measure of the amount of haemoglobin that is bound to oxygen compared to how much haemoglobin remains unbound in the blood. SpO_2_ refers to the saturation of peripheral oxygen reported as the percentage of oxygen in the blood. SpO_2_ values can be observed in individuals with heart and lung conditions, OSA and at high altitude^63^. Previously mostly used only in high-intensity hospital settings, COVID-19 led to much wider use of oximetry in the community, including monitors bought directly by citizens and patients. SpO_2_ can be measured using reflectance PPG in wearables^4^. For the majority of individuals, a normal SpO_2_ is between 95% and 99%.

In a 2023 review, five publications (*n* = 973) using Apple Watch Series 6 to measure SpO_2_ were evaluated (Table 2)^64^. When compared to medical-grade pulse oximeters, the 95% limits of agreement were reported to be −2.7% to 5.9% SpO_2_, however, outliers of up to 15% were reported. Whether wearables are suited for remote monitoring in patients with established conditions that affect their oxygen saturation is yet to be addressed and further validation in both patients and healthy controls is necessary before smartwatches are recommended for clinical use^65^. In a recent study using Garmin’s health snapshot, we observed frequent underestimation of SpO_2_^32^.

## Step count and distance travelled

Step count is a measure of physical activity which can objectively be measured by counting the number of steps an individual takes in a given period of time. The number of steps taken per day have strong associations with risk of chronic disease and mental health^66^ and are inversely related to obesity, OSA, gastroesophageal reflux disease and major depressive disorder^67^. The length of a walking step correlates to an individual’s height^68^, however, can be impacted by age, fitness level and health status.

Step count using wearables is derived as a composite of walking motion detected by the accelerometer and stride length (determined by pre-programmed height) and has been used as an outcome in clinical trials^69^. Distance travelled is commonly calculated from step count or measured through the activation of the GPS during outdoor activities and is the primary outcome of established sub-maximal tests of exercise capacity such as the 6 min walk test^70^.

32 studies of Garmin smartwatches were assessed for step count validity in a 2019 review^71^. 16 studies were found to have good (0.75–0.89) to excellent ( ≥ 0.90) correlation coefficients with acceptable APE ( < 5% in laboratory or controlled conditions and <10% in free-living conditions)^71^. Distance validity, which was tested in three studies, had lower correlation coefficients of <0.60 with acceptable APE and both over and underestimation was reported^71^.

In the context of established sub-maximal tests of exercise capacity, we recently observed that distance measured by Garmin and Fitbit through the activation of GPS was accurate, with as little as 6–8% error during a 6 min walk test if participants walked around a park^37^. However, error increased to 18–20% when a standard 30 m lap protocol was used. Step count was a more accurate measure of distance compared to GPS distance (MAPE: 0.9% [0.4, 2.2%] and 6.8% [3.2, 12.9%] for Garmin and Fitbit, respectively)^37^.

A 2020 systematic review of wearables (Fitbit, Apple Watch, Samsung and Garmin) also evaluated the reliability and validity of the measurement of step count^33^. From 158 studies, 805 comparisons between wearable derived step count and reference measures (manual counting or accelerometery) were made. Of these, 364 (45.2%) were within ±3% measurement error, 344 (42.7%) were below −3% measurement error and 97 (12.1%) were above 3% measurement error with an overall tendency to underestimate step count^33^.

## Physical Activity Recognition

Physical activity recognition can refer to the classification process of physiological motion measurements that may occur in a laboratory or free-living conditions^72^. Wearables often include a feature whereby the type and duration of a physical activity is automatically recognised and recorded without input from the user.

In 2019, a validation study assessed the automatic identification of physical activity type and duration using three Fitbit models (Flex 2, Alta HR and Charge 2) and one Garmin model (Vivosmart HR)^73^. The activities were a treadmill walk, treadmill run, embedded run, outdoor walk, outdoor run, elliptical, bike and swim, each for a duration of 15 min. The proportion of trials in which the activity type was correctly identified was 93% to 97% for treadmill walking, 93% to 100% for treadmill running, 36% to 62% for treadmill running when preceded and followed by a walk, 97% to 100% for outdoor walking, 100% for outdoor running, 3% to 97% for using an elliptical, 44% to 97% for biking, and 87.5% for swimming^73^.

## Cardiorespiratory Fitness (CRF) and VO_2_max Estimation

During exercise, an integrated and coordinated response from the heart, lungs, cardiovascular system and skeletal muscles is required to meet the metabolic demands of contracting muscles^74^. Maximal oxygen consumption (VO_2_max) is dependent on the ability of the oxygen transport system to deliver blood and the ability of cells to take up and utilise oxygen in energy production^75^. While maximal cardiopulmonary exercise testing (CPET) is the gold standard for assessing VO_2_max^76^, it requires clinical staff, space, expensive equipment and time and is rarely performed even in medical assessments.

CRF has been linked to several health-related outcomes, with low fitness being associated with increased risk of cardiovascular disease^77,78^, metabolic syndrome^79^, cognitive function^80^ and severe COVID-19^81^. Concurrently, increased levels of CRF are widely promoted as cardioprotective measures in the primary and secondary prevention of cardiovascular and coronary heart disease^82,83^ and a useful marker of training effectiveness in athletic individuals.

Smartwatch estimates of CRF can be estimated using anthropometric parameters (age, sex, height and weight), PPG-measured HR and HRV at rest, the relationship between changes in PPG-measured HR and HRV in relation to estimated workload during physical activity, exercise type or a combination of these factors using proprietary algorithms^84,85^.

A systematic review with meta-analysis of 14 studies that assessed the validity of smartwatch estimation of VO_2_max using either resting measurements (seated or supine resting HR) or exercise test-based measurements (outdoor running for at least 10 min) was performed in 2022 (Table 2)^86^. In the context of using resting measurements, an overestimation of VO_2_max was observed (mean difference [LoA]= 2.17 [−13.07, 17.41] ml/kg/min, *p* = 0.020) compared to the reference measurement. In contrast, a bias close to nil compared to the reference measurement (mean difference [LoA]= −0.09 [−16.79, 16.61] ml/kg/min, *p* = 0.910) was observed when outdoor running exercise measurements were used. However, the studies included in the meta-analysis were small (mean sample per study of 29) and based on young (pooled age 24.6 ± 5.7 years) healthy adults, who were active, recreational runners or soccer players^86^.

Absolute values and changes over time in VO_2_max as measured by Apple or Garmin wearables and CPET have been shown to correlate well (Pearson’s >0.80) in 48 adults with complex congenital heart disease^87^. However, in line with the findings of the Apple smartwatch validation study^49^ and prior work of ours utilising free-living activity^88^, despite observing moderate correlations, a large positive bias in smartwatch estimated VO_2_max has been observed, indicating that wearables often overestimate VO_2_max^87^.

Wearable estimates of CRF are currently limited to VO_2_max as an outcome parameter. In contrast, clinical CPET provides many other valuable metrics such as anaerobic threshold and oxygen uptake efficiency slope that contribute to a more comprehensive assessment of CRF and the utilisation of oxygen throughout exercise.

## Energy expenditure

Energy expenditure is defined as the energy expended, above resting levels, during purposeful exercise^89^. Doubly labelled water, is considered the gold-standard indirect calorimetry assessment of free-living energy expenditure^90^, however, is associated with high costs and limited by the requirement for structured activities in laboratory settings^91^. Wearables estimate energy expenditure, often referred to as ‘Calories burned’ or similar, using anthropomorphic data such as body mass, PPG-measured HR, physical activity derived from accelerometery, GPS or both and exercise intensity using proprietary algorithms.

Two systematic reviews established that commercially available wearables estimated energy expenditure with insufficient validity^33,92^. Consistent with a separate review of Fitbit accuracy^93^, it was reported that wearables tend to underestimate energy expenditure compared to criterion laboratory measures (Oxycon Mobile, CosMed K4b2, or MetaMax 3B), however, at higher intensities of activity energy expenditure is overestimated^92^.

In 2020, it was also reported that no brand of wearable was within ±3% of measurement error more than 13% of the time^33^. Underestimation of energy expenditure was observed in Garmin wearables 69% of the time, and in Withings wearables 74% of the time, respectively. Apple wearables overestimated energy expenditure 58% of the time and Polar wearables overestimated energy expenditure 69% of the time, respectively. Despite showing reasonable median value for accuracy, Fitbit devices underestimated energy expenditure 48% of the time and overestimated energy expenditure 40% of the time^33^.

## Blood Pressure (BP)

BP is the outward force by which blood pushes against the artery walls as it moves around the body. BP is described as the systolic over the diastolic BP (maximum over minimum) measured in millimetres of mercury (mmHg). Hypertension (elevated BP levels) affect more than 1 billion people globally and is the leading modifiable risk factor for preventable death^94^. The most commonly used method of BP assessment incorporates a cuff sphygmomanometer to assess brachial arterial BP level which can be incorporated into automatic oscillometric devices with a brachial cuff^95^.

Some wrist-worn devices (e.g., Omron HeartGuide) incorporate a cuff to measure BP at the wrist, but limited data on their accuracy is publicly available.

Cuffless wearables are emerging but often require user calibration prior to use^8^. BP measurements can then be derived from the time it takes for an arterial pulse wave to reach the periphery (pulse transit time) using ECG or pulse wave analysis using PPG in which the change in blood volume with each heart beat is assessed^96^. The amplitude of the PPG signal can provide information about the strength of the pulse, with consistently elevated amplitude in signal being an indication of hypertension^7^. The devices reviewed in Table 1 do not measure BP, but information about cuffless wearables for blood pressure monitoring can be found elsewhere^95,97^.

A systematic review and meta-analysis of 16 studies (*n* = 974) was performed in 2022^98^. 81% of devices in the analyses used PPG to estimate BP against a reference device. The authors defined devices with a mean bias of <5 mmHg as valid as a consensus. Eight devices showed a mean bias of <5 mmHg for SBP and DBP compared with a reference device, three of which were commercially available (B-Pro, Somnotouch-NIBP and T2-Mart). Differences were not observed between the wearables and reference devices for SBP (pooled mean difference = 3.42 mmHg, 95% CI: −2.17, 9.01) and DBP (pooled mean difference = 1.16 mmHg, 95% CI: −1.26, 3.58), however, confidence intervals around the estimates was wide. Recent data have cast doubt about the accuracy of some these devices^99,100^ and cuffless-based technology^101^, and our own data have shown limited agreement between cuffless and cuff-based ambulatory BP monitoring^102^.

## Sleep Duration and Stages

Sleep is an essential biological function with major roles in recovery, energy conservation and survival^103^. There is marked individual variation in the amount of sleep that an individual will need throughout the life span to ensure good health^104^. Objectively measured short and long sleep duration have been both associated with adverse health outcomes^105–107^.

The gold-standard assessment of sleep is laboratory-based polysomnography in which several parameters including brain waves, HR, respiratory rate, eye movement and muscle activity are monitored to classify sleep and wake cycles^108^. Wearables use a combination of PPG sensors and accelerometers to detect changes in HR and movement to calculate total sleep duration and classify sleep stages.

In 2023, a validation study was performed to assess the accuracy of 11 commercially available devices including five wearables (Google Pixel Watch, Galaxy Watch 5, Fitbit Sense 2, Apple Watch 8 and Oura Ring 3) compared to laboratory polysomnography in 75 participants^109^. Three wearables (Google Pixel Watch, Galaxy Watch 5 and Fitbit Sense 2) demonstrated moderate agreement with sleep stage classification (k = 0.4,0.6) and two wearables (Apple Watch 8 and Oura Ring 3) showed fair agreement (k = 0.2,0.4). The authors reported that wearables generally overestimate sleep by misclassifying periods of awake stillness as sleep. The Oura ring showed negligible proportional bias, potentially owing to its use of additional features beyond actigraphy such as body temperature and circadian rhythm for sleep staging^110^.

Fitbit devices were reported to be comparable to polysomnography in accuracy of detecting sleep phases, with 95% to 96% sensitivity and 58% to 69% specificity in detecting sleep epochs in a 2019 review^111^. A recent systematic review of eight studies investigated the accuracy of Fitbit, Garmin and Whoop in measuring sleep duration and sleep stages versus polysomnography^108^. Whoop was reported to have the least disagreement compared to polysomnography for total sleep time (−1.4 min), light sleep (−9.6 min) and deep sleep (−9.3 min) but the largest disagreement for rapid eye movement (REM) sleep (21.0 min). The Fitbit and Garmin devices both showed moderate accuracy in assessing sleep stages and total sleep time compared to polysomnography^108^.

## Psychological stress

Stress can be defined as a state of worry or mental tension caused by a challenging situation in life or the environment. Stress activates the sympathetic nervous system resulting in an increase in HR and a decrease in HRV^112^. Conversely, during periods of relaxation and sleep, a decrease in HR and increase in HRV can be observed^113^. Chronic stress, the repeated occurrence of the stress response over a period of time, is associated with increased risk of cardiovascular disease and type 2 diabetes mellitus^114,115^. The integration of stress management features in wearables is based on HR and HRV analysis and in some, EDA sensors. HRV analysis in wearables may be accompanied by real-time prompts to perform breathing activities or relaxation techniques in periods of detected stress.

Smartwatches were found to more accurately detect periods of psychological stress when utilising HRV and other physiological parameters such as EDA, respiratory rate and temperature instead of HR alone^116^. However, concerns surrounding the reliability of EDA measurement due to motion artifact have been raised^117^. Similarly, the accuracy of stress detection has been noted to decline during periods of vigorous movement, also likely owing to motion artifact^116^.

## Women’s Health

The monitoring of fertile windows and menstrual cycles have long been utilised for achieving planned pregnancy, preventing unplanned pregnancy, and the identification of irregular or abnormal cycles. Some wearables measure changes in HRV, body temperature and respiratory rate to detect and predict menstrual cycle stages including menstruation, the luteal phase and ovulation^118,119^.

A recent review of 13 studies found that most devices had high accuracy for detecting fertility and were able to differentiate between the luteal phase, fertile window and menstruation by changes in HR, HRV, temperature and respiratory rate (Table 2)^120^. In 2019, a study of the Oura ring reported a sensitivity for ovulation detection of 83.3% (−3 to +2 days) and menstruation detection of 71.9% to 86.5% (SD 2–4 days) using nocturnal finger skin temperature^119^. Compared to menstruation, a rise in HR (*p* = 0.001) and temperature (*p* < 0.001) during the luteal phases and lower HR (*p* = 0.02) and temperature (*p* = 0.05) during ovulation has been reported using the Oura ring more recently^118^.

## Data acquisition and analysis pipelines

When selecting a wearable device for clinical or research purposes, data accessibility is a crucial consideration. Prior to wearable device selection, we would recommend reviewing device specifications to ensure that the measurement parameters of interest (Table 1) are available (at the sampling frequency required), accessible and exportable in a usable format.

Access to raw data varies by manufacturer and model. While some devices display several measurement parameters to users, export options may be limited. No manufacturer currently allows for the export of continuously recorded raw signals (i.e., PPG, 3D accelerometer, temperature etc.), except for the ECG, which can often be exported as a series of 30-second long recordings. Most of the data that can be exported for off-line analysis consist in aggregate time-series summarising the trend of a physiological parameter with a temporal resolution that typically goes from one second (e.g., HR) to 15 min (e.g., number of steps or respiratory rate for some brands) to 1 day (e.g., sleep duration). Of note, no smartwatch or smart ring currently allows for the export of beat-to-beat HR time-series. Instead, instantaneous HR is usually averaged using undetermined filters, resulting in relatively smooth trends. Physiological parameters from physical activities recorded by the user (e.g. running, walking, cardio etc.) may include ad-hoc information (e.g., distance, speed, altitude etc.) with sometimes a better temporal resolution (e.g., HR provided every second instead of every minute), or they may be only exportable as summary statistics (e.g., total number of steps and average HR). As an example, Supplementary Table 1 provides a comprehensive list of parameters that can be exported from a Garmin smartwatch (VivoActive 4), along with their temporal resolution and whether they are passively recorded or may need user’s input. Additionally, some manufacturers have introduced premium or subscription-based models that may restrict data access.

The format of exported raw data files is another important consideration. Many manufacturers, including Fitbit, Oura and Withings, use standard file formats such as CSV or TXT, which are relatively easy to process. However, others, including Garmin, use more complex formats such as .FIT files for some of their data export, which require more advanced data processing skills to access.

To support data retrieval and management, open source platforms such as RADAR-base^121^, offer infrastructure to facilitate data storage and processing, and third-party services are becoming available^122,123^.

Data security and privacy should be considered owing to the sensitive nature of the personalised health information that these devices collect, particularly when activating GPS tracking systems for location information. To mitigate data security and privacy risks, manufacturer privacy settings and security policies should be reviewed ahead of device selection.

## Clinical Perspective for Cardiovascular Health

Cardiovascular diseases are the leading cause of death worldwide^124^. To reduce the enormous burden of cardiovascular diseases and disability^125^, progress is required in prevention, diagnosis, treatment and monitoring, and wearable data may play a useful role in all of these situations. The ability to collect data over months or years allows approaches that are not currently possible, however, may also present challenges in terms of the volume of data collected.

Evidence of the value of wearable data comes from the ability to detect abnormal heart rhythms, particularly AF, in people who would otherwise be unaware of this condition (and so at higher risk of stroke and other complications)^49,126^. Although questions remain about how to manage AF detected by such approaches, the ability to detect such a common and potentially devastating condition shows the potential for improved healthcare.

The addition of the ability to record a short ECG with sufficient accuracy to be approved as a medical device is now replacing more conventional ways to attempt to detect intermittent abnormal heart rhythms (such as issuing patients with such devices or recording the ECG for 72 h hoping to capture an event). Unlike PPG, ECG can only be recorded for short periods and often triggered by the user and so do not replace other methods to continually monitor ECG (such as Holter or implanted ECG recorders).

Arrhythmia can be diagnosed accurately using a single type of wearable data (ECG or HR characteristics from PPG). In contrast, other important cardiovascular diseases (such as heart failure, valvular heart disease, coronary artery disease, stroke) require specialist tests (such as cardiac ultrasound, coronary angiogram) to make an accurate diagnosis. However, the data wearables currently collect may still be useful. Heart failure and valvular heart disease are associated with increasing breathlessness on exertion and a reduced ability to be active^127^. It is highly likely that patterns of activity, respiratory rate, oximetry, and HR detected by wearables will change as a person develops these diseases and recent studies are encouraging^16,128^. Research is urgently required to test this potential, which may allow new approaches to community-based screening or diagnostic programmes.

Most patients who are diagnosed with cardiovascular disease typically have very limited interaction with healthcare services except when a crisis occurs (such as cardiac arrest, heart attack or urgent admission with heart failure). Such crises are often preventable if the signs of potential deterioration can be detected early enough for a change in management. Wearables hold great potential for such monitoring. For example, patients discharged after a heart attack are directed to adhere to a structured programme of physical activity^129^, and wearables have the potential to allow the healthcare system to know if patients are following such advice and target support to those who need this. People with heart failure suffer frequent worsening that can lead to life-threatening complications that are often preceded by weeks or months of increasing weight, reducing physical activity. These incipient signs of deterioration are likely also to be recognisable in changes in HR, respiratory rate, oximetry and BP. Wearables may therefore allow heart failure services to monitor patients and institute treatment (such as increasing diuretic therapy) to prevent admissions.

There remain several barriers to achieving the potential of wearables in healthcare. There is a pressing need for high-quality clinical studies that demonstrate the clinical scenarios in which providing wearable data improves outcomes. Data for its own sake is not helpful unless it can lead to an action that improves the patient’s health and clinicians already suffer “information overload”. Furthermore, in the health economic context, interventions need to meet a cost-benefit criteria.

Although consumer wearables are very attractive for introduction in healthcare due to their low cost and already high ownership levels, regulatory safeguards make this challenging. If data from wearables is used to make a clinical decision, then under current regulatory frameworks the wearable requires approval as a medical device. Most devices do not have such approvals and so using them in direct healthcare may pose legal risk.

## Population Health Perspective

The large-scale collection of health data using consumer-grade wearables has the potential to address pressing population health challenges, including the obesity epidemic, mental health epidemic, and the growing burden of chronic illnesses and multimorbidity. Wearables provide an objective continuous stream of data, offering a more comprehensive and possibly more reliable alternative to traditional population health approaches like self-reported questionnaires, which are limited by recall bias and infrequent administration. The widespread uptake of wearables and thus the availability of vast amounts of remote data may play a pivotal role in advancing population-based research, answering key health questions and developing risk stratification tools to better target interventions.

Many cardiovascular diseases are preventable by increasing physical activity, changing diet, maintaining a healthy weight and lowering BP^130^. Wearables are already used by millions of people to monitor their levels of physical activity. Although total amount of daily activity is known to be protective, recent evidence shows that even very short bursts of vigorous activity can provide a substantial reduction in risk^131^. There is evidence that tracking physical activity using wearable devices can provide a modest increase in total amount performed^132^. However, using such tools as a way to evaluate and refine the effect of more general public health measures (such as education, improved public transport, or work-place interventions) may improve the evidence base of how to facilitate healthier behaviours in the population.

During the COVID-19 pandemic, it was demonstrated via the ZOE COVID symptom app and Covid Collab Fitbit study that large-scale collection of digital health data was feasible and valuable^133,134^.

Limitations and risks associated with population level monitoring using wearables should also be considered. For example, it’s conceivable that being monitored continuously might alter clinical relationships which are based on trust or compromise mental health. Personalised approaches should be implemented with caution, ensuring that the responsibility or burden of blame for certain health behaviours is not unfairly positioned from society to the individual. Furthermore, consideration must be made for the cost associated with the provision of wearables and similarly, selection bias if study participants have been recruited based on prior device ownership or high level of digital literacy as there is a risk of exacerbating health gradients along the digital divide, and excluding from interventions those strata that may receive the greatest benefit.

## Supplementary information

Supplementary information

## Data Availability

No datasets were generated or analysed during the current study.
