# Supplementary material for: A guide to consumer-grade wearables in cardiovascular clinical care and population health for non-experts
Source: NPJ Cardiovasc Health. 2025 Sep 2;2:44. doi: 10.1038/s44325-025-00082-6 (PMC12404996; doi:10.1038/s44325-025-00082-6)
Supplement: Supplementary file 1 — Supplementary information [file 44325_2025_82_MOESM1_ESM.pdf]

Supplementary Table 1: Comprehensive list of health and physical activity parameters from Garmin Vivoactive 4 as retrieved using the Radar Base platform. Sampling rate: Number of measurements within a data-file. Frequency: Number of times a data file is generated

PARAMETER	SAMPLING RATE	FREQUENCY
**ACTIVITY SUMMARY (IT REQUIRES THE USER TO INITIATE A RECORDING)**		
activeKilocalories	single number	One per recorded activity
activityType	single number	One per recorded activity
averageHeartRate	single number	One per recorded activity
averagePace	single number	One per recorded activity
averageRunCadence	single number	One per recorded activity
averageSpeed	single number	One per recorded activity
distance	single number	One per recorded activity
duration	single number	One per recorded activity
maxHeartRate	single number	One per recorded activity
maxPace	single number	One per recorded activity
maxRunCadence	single number	One per recorded activity
maxSpeed	single number	One per recorded activity
startingLatitude	single number	One per recorded activity
startingLongitude	single number	One per recorded activity
steps	single number	One per recorded activity
time	single number	One per recorded activity
totalElevationGain	single number	One per recorded activity
totalElevationLoss	single number	One per recorded activity
**ACTIVITY DETAILS (IT REQUIRES THE USER TO INITIATE A RECORDING)**		
clockDuration	1 second	One per recorded activity
elevation	1 second	One per recorded activity
heartRate	1 second	One per recorded activity
latitude	1 second	One per recorded activity
longitude	1 second	One per recorded activity
movingDuration	1 second	One per recorded activity
speed	1 second	One per recorded activity

time	1 second	One per recorded activity
timerDuration	1 second	One per recorded activity
totalDistance	1 second	One per recorded activity
**BODY BATTERY**		
bodyBattery	3 minutes	Daily
time	3 minutes	Daily
**PHYSICAL ACTIVITY - DAILY SUMMARY (PASSIVE RECORDINGS)**		
activeKilocalories	single number	Daily
activeTime	single number	Daily
activityStressDuration	single number	Daily
activityType	single number	Daily
averageHeartRate	single number	Daily
averageStressLevel	single number	Daily
bmrKilocalories	single number	Daily
date	single number	Daily
distance	single number	Daily
duration	single number	Daily
floorsClimbed	single number	Daily
floorsClimbedGoal	single number	Daily
highStressDuration	single number	Daily
intensityDurationGoal	single number	Daily
lowStressDuration	single number	Daily
maxHeartRate	single number	Daily
maxStressLevel	single number	Daily
mediumStressDuration	single number	Daily
minHeartRate	single number	Daily
moderateIntensityDuration	single number	Daily
restStressDuration	single number	Daily
restingHeartRate	single number	Daily
steps	single number	Daily
stepsGoal	single number	Daily
stressDuration	single number	Daily
stressQualifier	single number	Daily
time	single number	Daily

vigorousIntensityDuration	single number	Daily
**PHYSICAL ACTIVITY - TIME SERIES (PASSIVE RECORDINGS)**		
activeKilocalories	15 minutes	Daily
activeTime	15 minutes	Daily
activityType	15 minutes	Daily
distance	15 minutes	Daily
duration	15 minutes	Daily
intensity	15 minutes	Daily
maxMotionIntensity	15 minutes	Daily
meanMotionIntensity	15 minutes	Daily
metabolicEquivalentOfTask	15 minutes	Daily
steps	15 minutes	Daily
time	15 minutes	Daily
**HEART RATE - TIME SERIES**		
heartRate	15 seconds	Daily
time	15 seconds	Daily
**AUTOMATIC ACTIVITY DETECTOR**		
activitySubType	Automatic	Automatic
activityType	Automatic	Automatic
date	Automatic	Automatic
duration	Automatic	Automatic
time	Automatic	Automatic
**PULSE OXIMETRY**		
date	1 min	Daily
duration	1 min	Daily
spo2Value	1 min	Daily
time	1 min	Daily
**RESPIRATION**		
duration	1 min	Daily
respiration	1 min	Daily
time	1 min	Daily
**SLEEP - TIME-SERIES**		
endTime	3 minutes	Daily
sleepLevel	3 minutes	Daily

startTime	3 minutes	Daily
time	3 minutes	Daily
**SLEEP - DAILY SUMMARY**		
awakeDuration	single number	Daily
date	single number	Daily
deepSleepDuration	single number	Daily
duration	single number	Daily
lightSleepDuration	single number	Daily
remSleepDuration	single number	Daily
time	single number	Daily
**STRESS - DAILY SUMMARY**		
date	single number	Daily
duration	single number	Daily
time	single number	Daily
**STRESS - TIME SERIES**		
stressLevel	3 minutes	Daily
time	3 minutes	Daily
**FITNESS**		
date	Automatic	Automatic
fitnessAge	Automatic	Automatic
time	Automatic	Automatic
vo2Max	Automatic	Automatic
**BODY COMPOSITION**		
weight	Manual	Manual
